# The human decapping scavenger enzyme DcpS modulates microRNA turnover

**DOI:** 10.1038/srep16688

**Published:** 2015-11-20

**Authors:** Oussama Meziane, Sandra Piquet, Gabriel D. Bossé, Dominic Gagné, Eric Paquet, Claude Robert, Michael A. Tones, Martin J. Simard

**Affiliations:** 1 St-Patrick Research Group in Basic Oncology, Centre Hospitalier Universitaire de Québec-Université Laval (Hôtel-Dieu de Québec), Laval University Cancer Research Centre, Quebec City, Québec, Canada; 2 Laboratoire de génomique fonctionnelle du développement embryonnaire, Centre de recherche en biologie de la reproduction, Pavillon Comtois, Faculté des sciences de l’agriculture et de l’alimentation, Université Laval, Quebec City, Québec, Canada; 3 Rare Disease Research Unit, Pfizer, 610 Main Street, Cambridge, Massachusetts, USA

## Abstract

The decapping scavenger enzyme DcpS is known for its role in hydrolyzing the cap structure following mRNA degradation. Recently, we discovered a new function in miRNA degradation activation for the ortholog of DcpS in *C. elegans*. Here we show that human DcpS conserves its role in miRNA turnover. In human cells, DcpS is a nucleocytoplasmic shuttling protein that activates miRNA degradation independently of its scavenger decapping activity in the cytoplasmic compartment. We also demonstrate that this new function for DcpS requires the contribution of the 5′-3′ exonuclease Xrn2. Our findings support a conserved role of DcpS as a modulator of miRNA turnover in animals.

MicroRNAs (miRNAs) are small non-coding RNAs regulating protein production upon binding 3′UTR of its target mRNA by partial complementary sequence interaction inducing translation inhibition and/or mRNA degradation. The miRNA-mediated gene regulation mechanism is conserved in nearly all eukaryotes including humans. It is predicted that miRNAs could regulate expression of at least 30% of human genome, implicating them in the regulation of almost all biological processes (Reviewed in^1^). Consequently, miRNA levels alteration observed in human diseases supports their importance in cell homeostasis (reviewed in^2^,^3^). It is therefore essential to understand the molecular mechanisms controlling miRNAs biogenesis and stability.

MiRNA genes are mostly transcribed by RNA polymerase II to form a miRNA primary transcript (pri-miRNA). In the nucleus, the pri-miRNA is processed to produce the miRNA precursor (pre-miRNA) by a microprocessor complex containing the RNase III-like enzyme Drosha and the RNA binding protein DGCR8 to produce the miRNA precursor (pre-miRNA). After its export to the cytoplasm, the pre-miRNA is cleaved by another RNase III-like enzyme, Dicer, to produce the mature miRNA. Subsequently, the miRNA is bound to an Argonaute protein to form the miRNA Induced Silencing Complex (miRISC). The miRNA serves as guide for the miRISC to target specific mRNA, inducing inhibition of protein production by different ways (Reviewed in^4^). Over the years, several mechanisms responsible for either activating or blocking the biogenesis of a specific miRNA or a subset of miRNAs in response to different cellular conditions were uncovered (Reviewed in^5^,^6^). Nevertheless, the fate of mature miRNAs after binding to mRNAs remains unclear. Recently, the involvement of some ribonucleases in the degradation of specific mature miRNAs has been reported in plants (SDNs), *Caenorhabditis elegans* (XRN-1 and XRN-2), and human cells (Xrn1, PNPase and RRP4)^7^.

Recently, we have identified DCS-1, the ortholog of the human decapping scavenger enzyme DcpS, as a new modulator of miRNA turnover in *Caenorhabditis elegans*. DCS-1 interacts with the 5′-3′ exonuclease XRN-1 to promote microRNA degradation independently of its known decapping activity^8^. To determine if the modulation of miRNAs turnover by DCS-1 is conserved in mammals, we surveyed the implication of human DcpS in miRNA stability. In this study, we report that the cytoplasmic DcpS activates miRNAs degradation in human cells with the contribution of the 5′-3′ exonuclease Xrn2. As observed in nematode, this process is independent of the decapping activity of DcpS. Our findings support a conserved role of DcpS as a modulator of miRNA turnover in animals.

## Results

### The inhibition of DcpS alters miRNAs degradation

**Figure 1:**
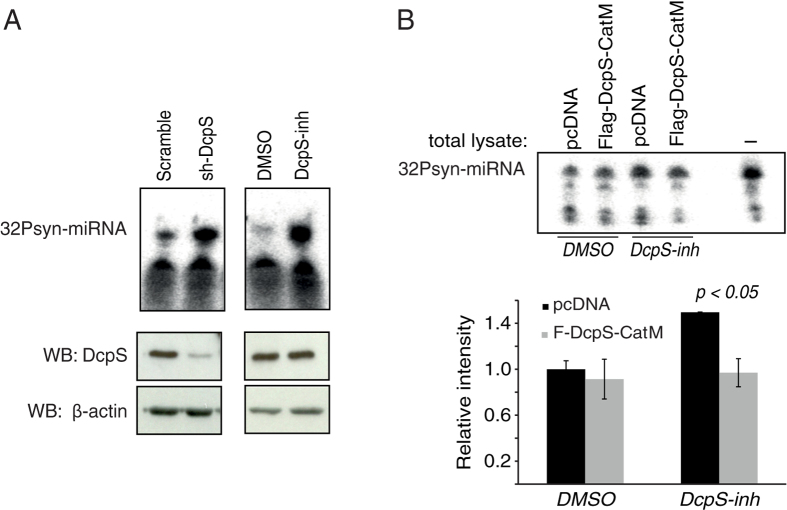
DcpS activates miRNA degradation independent of its decapping scavenger activity. (**A**) Inhibition of DcpS alters *in vitro* miRNA degradation. 10 μg of total protein extracts from cells either infected with lentivirus encoding for an shRNA targeting DcpS (left panels) or treated with a DcpS inhibitor (DcpS-inh; right panels) were incubated for 15 minutes with 5′-^32^P-labeled small RNA oligonucleotide that corresponds to let-7 miRNA sequence (32Psyn-miRNA). Knockdown of DcpS was validated by Western blotting (bottom panels). The endogenous β-actin was probed and used as loading control. (**B**) Catalytically inactive DcpS rescues miRNA degradation in cell treated with DcpS-inhibitors. 5′-^32^P-labeled synthetic miRNA (32Psyn-miRNA) was incubated for 10 minutes with 10 μg of total protein extracts from cells treated. First, cells were treated with DcpS-inh or DMSO and 6 h after were transfected with Flag tagged catalytically inactive DcpS mutant (Flag DcpS-CatM) expressing vectors or pcDNA vectors. Cells were harvested and proteins were lysed 12 h after transfection using RIPA buffer. The graph represents the quantification of three independent experiments and the error bars represent standard errors. Significance was analyzed using Student’s t-test.

To determine whether DcpS is important for miRNA stability in human cells, we first performed miRNA degradation assays *in vitro* using total protein lysates. While we observed a nearly complete degradation of a radiolabeled synthetic miRNA after incubation with lysate prepared from cell treated with non-targeting shRNA (scramble), the RNAi knockdown of DcpS severely impaired miRNA degradation (sh-DcpS; Fig. 1A left panel). In order to further confirm the direct implication of DcpS in miRNA degradation, we treated the cells with a compound that binds to the closed active site conformation of dimer DcpS and inhibits its activity^9^. As observed with DcpS RNAi, the treatment of cells with D156844 compound (DcpS-inh), at a concentration sufficient to abolish DcpS decapping activity (Figure S1), altered miRNA degradation efficiency when compared to cells treated with DMSO (Fig. 1A right panel). We therefore conclude that functional human DcpS is required for miRNA degradation *in vitro*.

### DcpS enhances miRNA degradation independently of its scavenger decapping activity

DcpS uses an evolutionarily conserved Histidine Triad (HIT) motif that confers a dinucleoside triphosphate hydrolase activity allowing the processing of the m7G cap structure remaining on the last nucleotides following the mRNA degradation^10^. In *C. elegans*, the ortholog of DcpS activates miRNA degradation independently of its decapping scavenger activity^8^. In order to test whether the DcpS-mediated miRNA degradation observed in human cells is also independent of its enzymatic activity, we introduced mutations within the HIT motif to abolish its activity (Figure S2). We observed that while the expression of the DcpS catalytic mutant (DcpS-CatM) does not rescue the decapping activity after treatment with the DcpS-inhibitor (Figure S1), its addition to cells treated with the inhibitor rescued miRNA degradation *in vitro* (Fig. 1B). We thus conclude that in human cells, as observed in *C. elegans*, the implication of DcpS in miRNA turnover is uncoupled from its *bona fide* decapping scavenger activity.

### The cytoplasmic but not nuclear DcpS activates miRNA degradation

**Figure 2:**
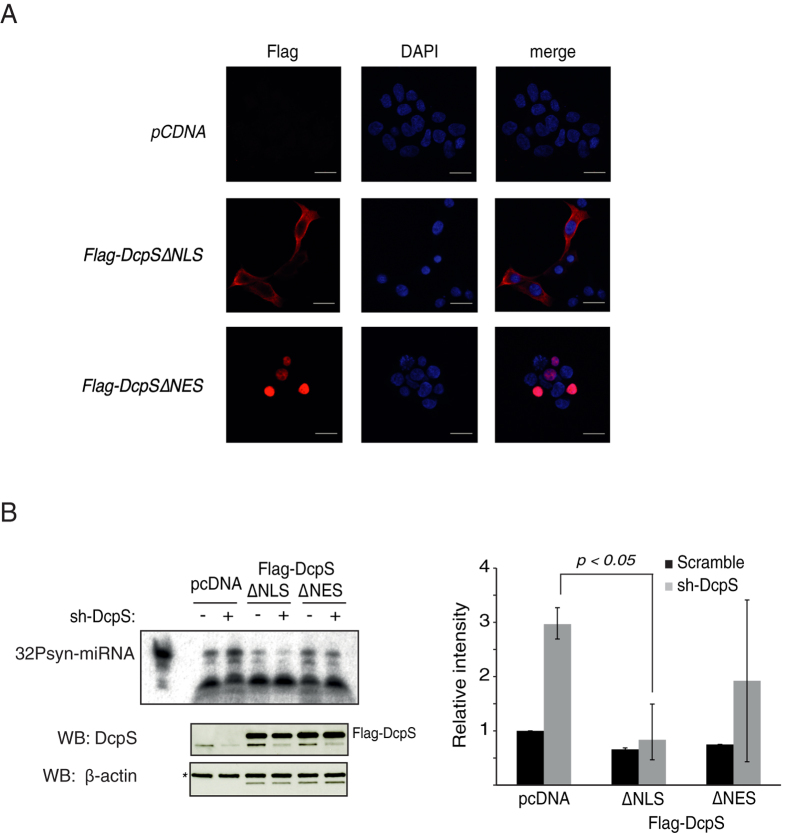
Cytoplasmic but not nuclear DcpS rescues miRNA degradation *in vitro*. (**A**) Localization of Flag-∆NLS-DcpS and Flag-∆NES-DcpS mutants detected by immunofluorescence using the anti-Flag M2 antibody (red). Dapi staining (Blue) represents nucleus. Cells transfected with the empty vector (pcDNA 3.1-Flag) are shown. (**B**) Cytoplasmic but not nuclear DcpS activates *in vitro* miRNA degradation. 5′-^32^P-labeled synthetic miRNA (32Psyn-miRNA) was incubated for 10 minutes with 10 μg of total protein extracts from cells infected with lentivirus encoding for either an shRNA targeting 3′UTR of endogenous DcpS or control shRNA (scramble) and transfected with vectors expressing either Flag tagged DcpS mutated for nuclear localization signal (Flag-DcpSΔNLS) or Flag tagged DcpS mutated for nuclear export signal (Flag-DcpSΔNES). Cells transfected with pcDNA 3.1-Flag empty vectors were used as control. Knockdown of endogenous DcpS (lower band) and overexpression of mutants (upper band) were confirmed by western blotting (bottom panel). The endogenous β-actin (marked with *) was probed and used as loading control (the lower band correspond to Flag-DcpS). The graph represents the quantification of three independent experiments. The error bars represent standard errors and significance was analyzed using a Student’s t-test.

In contrast to *C. elegans* DCS-1 which is exclusively cytoplasmic, human DcpS can be located in both the nucleus and the cytoplasm^9^,^11^,^12^. To identify in which cellular fraction DcpS induces miRNA degradation, we generated DcpS variants, carrying mutations abolishing either the nuclear localization signal or the nuclear export signal motifs (ΔNLS and ΔNES, respectively; Figure S2). As previously reported^13^, mutations in either motifs alter the localization of DcpS in cells (Fig. 2A). In order to determine in which cellular compartment DcpS activates miRNAs degradation, we first knockdown the endogenous DcpS using lentivirus producing shRNA targeting DcpS mRNA 3′UTR followed by the transfection of plasmids expressing Flag-tagged DcpS-∆NLS or Flag-tagged DcpS-∆NES mutants. The degradation assays revealed that the cytoplasmic DcpS variant, but not the nuclear one, re-established degradation of a synthetic miRNA in total protein lysate (Fig. 2B). We therefore conclude that the cytoplasmic fraction of DcpS contributes to the turnover of miRNAs observed in human cells.

### Xrn2 contributes to DcpS-dependent miRNA turnover in human cells

**Figure 3:**
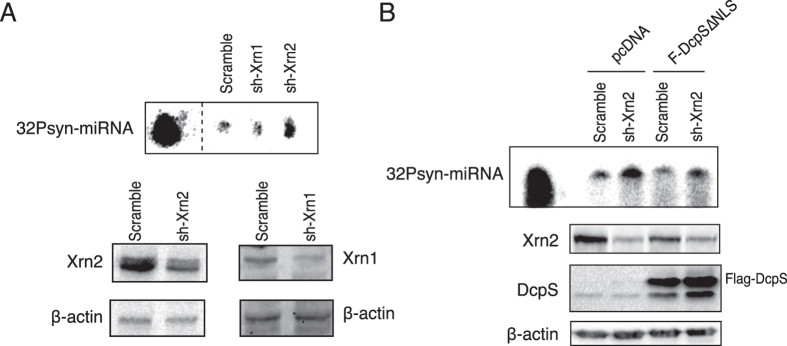
The 5′-3′ exonuclease Xrn2 contributes to DcpS-dependent miRNA degradation. (**A**) The knockdown of Xrn2 but not Xrn1 abrogates miRNA degradation *in vitro*. 5′-^32^P-labeled synthetic miRNA (32Psyn-miRNA) was incubated for 15 minutes with 10 μg of total protein extracts from cells infected with lentivirus encoding for shRNA targeting either Xrn1, Xrn2 or control shRNA (scramble). The efficacy of knockdowns was confirmed by western blotting (bottom panel). Dashed lines indicate that unrelated lanes have been removed between samples. (**B**) DcpS requires Xrn2 to support miRNA degradation. Cells were infected with shRNA targeting Xrn2 followed by transfection of plasmids expressing Flag-DcpS-∆NLS. Transfection with pcDNA 3.1-Flag empty vector served as control. Degradation assay was performed using 10 μg of total protein and incubated with a 5′-^32^P-labeled synthetic miRNA (32Psyn-miRNA) for 10 min. The efficacy of knockdown was validated by western blotting and the endogenous β-actin was probed and used as loading control (Bottom panel).

Since DcpS does not have any exonuclease activity by itself^14^, it thus needs the contribution of an exonuclease to promote miRNA turnover. In *C. elegans*, the conserved 5′-3′ exonucleases XRN-1/Xrn1 and XRN-2/Xrn2 have been implicated in miRNA degradation^8^,^15^. We therefore decided to determine whether human Xrn1 or Xrn2 contributes directly to miRNA degradation. We performed the degradation assays using total protein lysates from cell infected with shRNA lentiviruses targeting either Xrn1, Xrn2 or non-targeting any known mRNA (control scramble). While we did not observe any effect on miRNA degradation activity in cells extracts expressing the control and Xrn1 shRNAs, knockdown of Xrn2 significantly reduced miRNA degradation (Fig. 3A). To further confirm that the DcpS-inducing miRNA degradation is mediated by Xrn2, we overexpressed DcpS-∆NLS tagged construct in Xrn2 knockdown cells. While the overexpression of the cytoplasmic DcpS-∆NLS increased degradation of miRNA in control cells, the knockdown of Xrn2 abolished this activity (Fig. 3B). We next tried to investigate by immunoprecipitation whether the exonuclease can form a complex with DcpS in human cells to stimulate miRNA degradation as observed in *C. elegans*^8^. Despite several attempts with different variations in binding conditions (including cross-linking, changing salt concentrations for binding and washes), we have been unable to detect a stable interaction between Xrn2 and DcpS (data not shown). Taken all together, these data support that the exonuclease Xrn2 contributes with DcpS to control miRNA turnover in human cells.

### The inhibition of DcpS alters miRNA level in human cells

**Figure 4:**
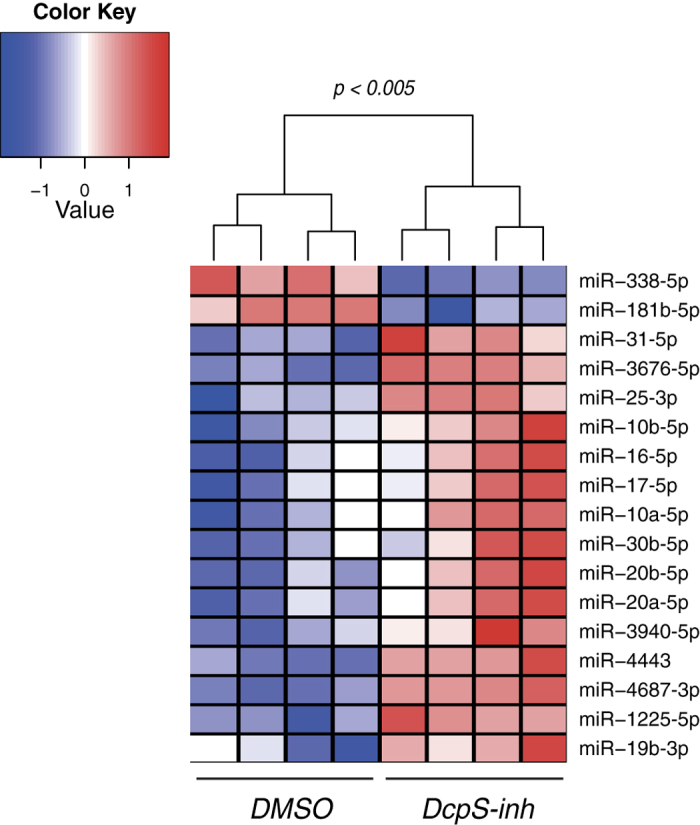
The inhibition of DcpS affects miRNA levels in human cells. miRNA profiles were measured by microarrays from cell treated with 500 nM of DcpS inhibitor (DcpS-inh). Heatmap presenting the supervised clustering of DMSO and DcpS-inh significantly differentially expressed miRNAs in HEK293T cells. The colorcode represents normalized expression values and blue, white and red correspond to low, intermediate and high expression respectively. The clustering is done using euclidean distance and complete agglomerative.

To determine whether the involvement of DcpS in miRNA degradation can lead to an alteration of endogenous miRNA levels, we quantified miRNAs in HEK293T cells after treatment with DcpS-inh. A microarray analysis indicated that among the 117 miRNAs detectable in HEK293T cells, 15 of them are significantly increased in cells treated with the DcpS-inhibitor relative to cells treated with DMSO whereas only two miRNAs decreased in treated cells (a percentage of downregulated miRNAs (1.7%) that is lower than the threshold for multiple testing correction; Fig. 4). We further validated the effect of the inhibition of DcpS on miRNA levels by performing TaqMan quantitative PCR assays for a representative number of affected miRNAs (Figure S3). To confirm that the increased miRNA levels upon DcpS inhibition are not caused by a modification of transcripts levels as recently reported for a subset of RNAs^16^, we measured the levels of a primary miRNA molecule coding for six different miRNAs (the miR-17 ~ 92 cluster) including miR-17, miR-19b and miR-20a, three miRNAs that are significantly increased in treated cells. Using quantitative PCR, we did not detect any significant changes in the level of the primary molecule between control DMSO and DcpS inhibitor treated cells (Figure S4A). In addition, we also examined the levels of miR-20a precursor molecules and did not detect any significant change upon DcpS inhibition (Figure S4B), suggesting that the DcpS inhibitor does not affect the biogenesis of miRNAs. We thus conclude that the inhibition of DcpS affects the stability of mature miRNA molecules in human cells.

## Discussion

Overall, our observations demonstrate that the DcpS function in miRNA turnover originally observed in nematodes is conserved in human cells. DcpS is a shuttling protein that scavenges residual cap structures produced as the result of mRNAs degradation in cytoplasm and is implicated in splicing regulation in the nucleus compartment^11^,^13^. Taken together, our data suggest a new cytoplasmic function of DcpS in miRNA turnover independent of its catalytic activity.

At the moment, it has been reported that Xrn1 and RRP41 regulate miR-382 stability in HEK293T after 8 h actinomycin D treatment^17^ and PNPaseold-35 modulates specific miRNA levels in human melanoma cells^18^. However, in our study we have identified a new mechanism implicating the exonuclease Xrn2 in miRNA turnover in human cells. Interestingly, using cellular fractions, a recent study reported that Xrn2 can also localize to the cytoplasm^19^. Our preliminary observations made by immunostaining also indicated the presence of a fraction of Xrn2 protein in the cytoplasm (data not shown). While the molecular mechanism activating a specific exonuclease for the degradation of a subset of miRNAs is still unclear, our data suggest the implication of DcpS in the specificity of Xrn2 to target miRNAs, potentially through a transient interaction between both proteins occuring in the cytoplasm.

Several studies have observed changes in the miRNA profile in different diseases such as cancer. A deregulation of DcpS expression as well as mutations could be a potential cause of the variation in miRNAs profiles observed in cancer. Thus, in our study we used a chemical inhibitor of DcpS to specifically inhibit its activity. Interestingly, treatment of different Spinal Muscular Atrophy (SMA) mice models with an analog of DcpS inhibitor improves surviving and motor functions^20^,^21^. While the therapeutic use of altering DcpS function to treat SMA patients is quite promising, it is still unclear molecularly how this inhibition can specifically acts on different SMA phenotypes. It has been shown that inactivating processing of miRNAs in spinal motor neurons in mouse models creates hallmarks of SMA^22^. Interestingly, miRNA profiling in different mice SMA models carrying mutation in the SMN gene revealed that some miRNAs are significantly down-regulated^22^. Based on our discoveries, it is therefore plausible that inhibiting DcpS in SMA mice models re-establishes the levels of specific miRNAs that are essential for spinal motor neurons.

## Methods

### Plasmid constructs

DcpS mutants were cloned in pcDNA3.1-Flag plasmid. The mutations sites were referred to Shen *et al.*^13^ (Figure S2).

### Cell culture and DcpS-inh treatment

HEK293T cells were grown in Dulbecco modified Eagle medium (DMEM) supplemented with 10% of fetal bovine serum (FBS) and 5% of Penicillin/Streptomycin at 37 °C in 5% CO_2_. 3 × 10^5^ cells were seeded in 6-well plates. Medium was changed at Day 2 with the addition of 500 nM final of D156844 DcpS-inh or DMSO (as perfomed by Singh *et al.*^9^). 18 h after treatment, cells were harvested and RNA or proteins were extracted using Absolutely RNA miRNA kit (Agilent Technologies) and RIPA buffer, respectively.

### shRNA lentivirus production and cell transduction

ShRNA was purchased from Sigma-Aldrich: TRCN0000005569 reference number for shRNA against 3′UTR of DcpS, TRCN0000296739 for Xrn1 shRNA, TRCN0000349677 for Xrn2 shRNA and SHC002 Non-Mammalian sh Control called ≪Sh Scramble≫. 2.5 × 10^6^ HEK293T cells were seeded in 10 mm petri. At day 2, cells were co-transfected with 2.5 μg of envelope plasmid pMD.2G encoding the G protein of vesicular stomatitis virus (VSV-g), 5 μg of psPAX2 plasmid and 5 μg of shRNA using Calcium phosphate transfection protocol provided by the manufacturer (Sigma-Aldrich). Two days after transfection, the medium containing the shRNA lentiviruses was collected and filtered using 45 μm filters.

HEK293T were transduced for 3 h at 37 °C, washed one time and maintained in culture with a fresh medium. At day 3, cells were transfected with different mutants using calcium phosphate transfection protocol and cells were collected 24 hours post-transfection.

### Immunofluorescence

2 × 10^5^ HEK293T cells were grown on polyK coated 35 mm plates and transfected with 500 μg of either pDNA3.1-Flag, pCDNA3.1-Flag-DcpS pCDNA3.1-Flag-ΔNES or pCDNA3.1-Flag-ΔNLS. 24 h after transfection, cells were fixed with PBS-PFA 4% (w/v). After washing with PBS, permeabilization was performed using PBS- triton X-100 0.5% (v/v) for 5 min, and then washed with PBS. After blocking in PBS-BSA 5% (w/v) for 20 min, anti-Flag M2 (Sigma; dilution 1:500 in PBS-BSA 5%) was incubated for 60 min, and then washed three times with PBS-tween 0.2% (v/v). Secondary Goat anti-Mouse IgG secondary antibody, Alexa Fluor^®^ 568 conjugate (Life Technologies. dilution 1:250 in PBS-BSA 5%) was incubated for 60 min, and after three washes, cells were mounted with 1 ug/ml DAPI staining.

### Decapping assay

Cap-labeled substrate RNAs were prepared from uncapped RNA using ^32^P-α-GTP (Perkin-Elmer) and Capping Vaccinia kit from New England Biolabs. The labelling reaction was performed following manufacturer’s protocol. Cap-labeled substrates were gel purified and incubated with 10 μg of total cell lysates (prepared using RIPA buffer with Cocktail EDTA-free Protease inhibitors (Roche)) for 2 or 5 min at 30 °C and loaded onto thin layer chromatography plates. Dried plates were exposed to image plates and than scanned with FLA-5100 phosphoimager (Fuji Photo Film Company). Images were analyzed using ImageJ.

### Degradation assay

Assays were performed as described previously in^15^. Briefly, an RNA oligonucleotide corresponding to let-7 miRNA sequence was 5′ radiolabeled with PolyNucleotide Kinase and γ-^32^P ATP and gel purified. 20 fmol of ^32^P labeled synthetic miRNA was incubated at 37 °C with 10 μg of total protein lysate in 1x of assay buffer (10 mM HEPES PH 7.4, 2 mM DTT, 5 mM MgCl_2_, 100 mM KCl, 2 mM ATP) in a final volume of 20 μl. The reaction was stopped by adding 1 volume of denaturating gel loading buffer (containing 95% (v/v) Formamide, 0.2% (w/v) SDS, 1 mM EDTA) followed by incubation at 90 °C for 1 min. the total volume of each sample were then loaded onto 10% denaturing polyacrylamide gels. Gels were dried, exposed to image plates and than scanned with FLA-5100 phosphoimager (Fuji Photo Film Company). Images were analyzed using ImageJ.

### miRNAs quantification

Total RNA, including miRNAs, was isolated with Absolutely RNA miRNA Kit (Agilent Technologies) according to the manufacturer’s protocol. RNA concentration, purity and RNA integrity number (RIN) were determined on an Agilent 2100 Bioanalyzer and RNA 6000 NanoLabChip Kits (Agilent Technologies). Only samples with a RIN equal or superior to 8.5 were selected for microarray hybridization.

MiRNA profiles of four non-treated and four treated samples were evaluated using the Unrestricted Human miRNA V19.0 microarray (8 × 60 K; Agilent Technologies). Briefly, 100 ng of total RNA were treated with alkaline calf intestine phosphatase and labeled with Cyanine 3-pCp. Labelled samples hybridized at 55 °C for 20 h at 20 rpm. After washing, the fluorescent signal intensities were detected on a PowerScanner (Tecan, Mannedorf, Switzerland) and analyzed with Array-Pro Analyzer software (MediaCybernetics, Bethesda, MD, USA). To evaluate the efficiency of labeling and hybridization, Agilent microRNA Spike-In were added in the appropriate steps.

Agilent miRNA expression arrays were processed using the package limma in Bioconductor^23^. Briefly, raw gene expression data were background subtracted and quantile normalized. The Agilent miRNA arrays have several copies (>10) of the same probes so we took the average of all the probes present on the array for downstream analyses. We considered a miRNA to be expressed in the DMSO treated condition if the per array expression value is higher than the 90th percentile value of the negative control probes present on the array. We detected significant difference in miRNA expression using an empirical bayes version of the t-test implemented in limma^23^. We considered a miRNA to be significantly modulated using a cutoff of P < 0.005. Heatmaps were generated using Euclidian distance and a complete agglomerative method. All expression data have been submitted to the Gene Expression Omnibus (GEO) with accession number GSE69591.

The detection of microRNA levels by quantitative RT-PCR was performed using TaqMan small RNA assays (Life Technologies) following the manufacturer’s protocol. The miR-33b miRNA was used as control.

### Northern Blotting

Northern hybridizations were performed as described previously^8^.

## Additional Information

**How to cite this article**: Meziane, O. *et al.* The human decapping scavenger enzyme DcpS modulates microRNA turnover. *Sci. Rep.* **5**, 16688; doi: 10.1038/srep16688 (2015).

## Supplementary Material

Supplementary Information
