# Supplementary material for: The human decapping scavenger enzyme DcpS modulates microRNA turnover
Source: Sci Rep. 2015 Nov 20;5:16688. doi: 10.1038/srep16688 (PMC4653633; doi:10.1038/srep16688)
Supplement: Supplementary Information [file srep16688-s1.pdf]

The human decapping scavenger enzyme DcpS modulates microRNA turnover

Oussama Meziane, Sandra Piquet, Gabriel D. Bossé, Dominic Gagné, Eric Paquet, Claude Robert, Michael A. Tones and Martin J. Simard

SUPPLEMENTARY FIGURES

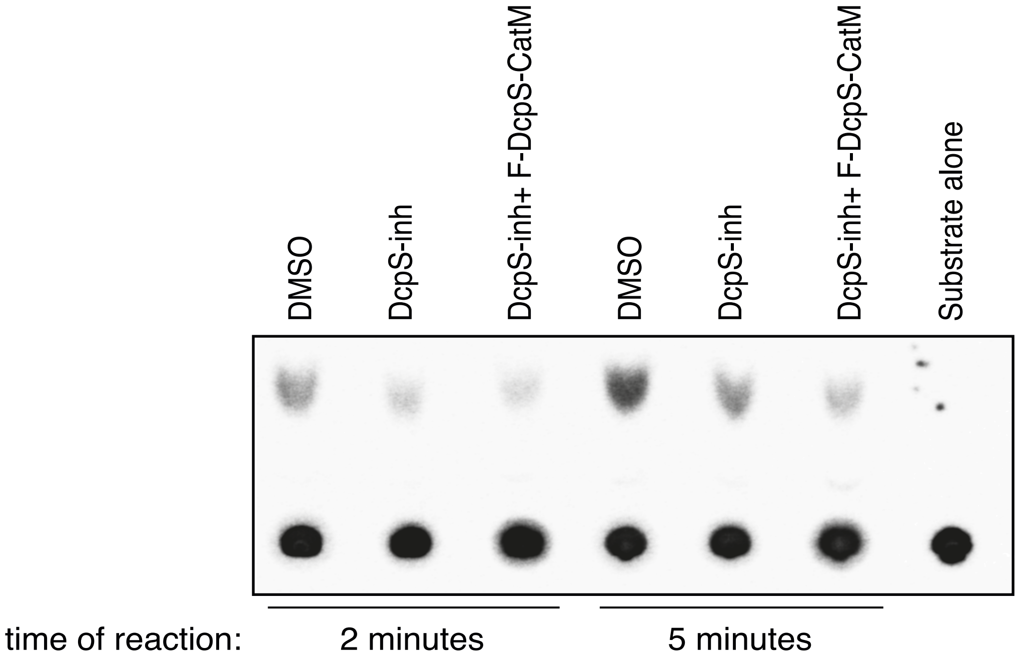

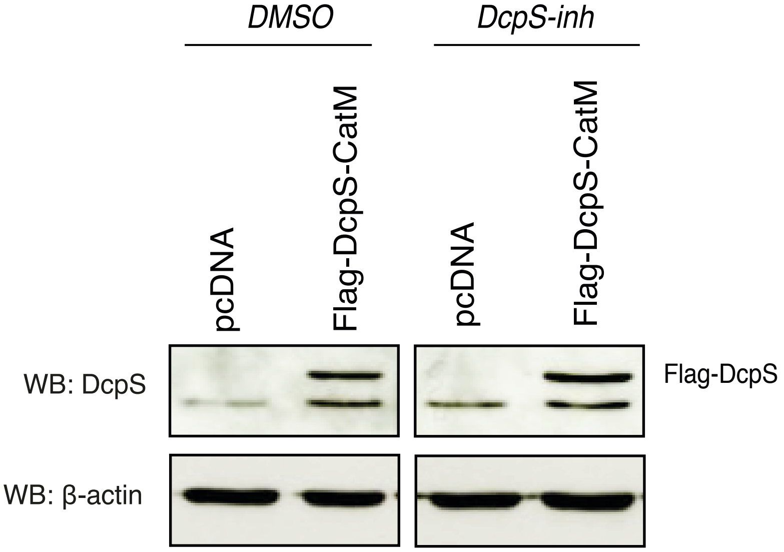

Figure S1: DcpS inhibitor D156844 abolishes the decapping scavenger activity of DcpS. Radiolabeled m7Gp*ppG was incubated with total protein extracts from cells 18h after treated with 500nM of DcpS-inh or with DMSO. The reaction products were separated by thin layer chromatography and visualized with a Phosphorimager. The expression of endogenous and Flag-DcpS-CatM were detected by western blotting (bottom panel).

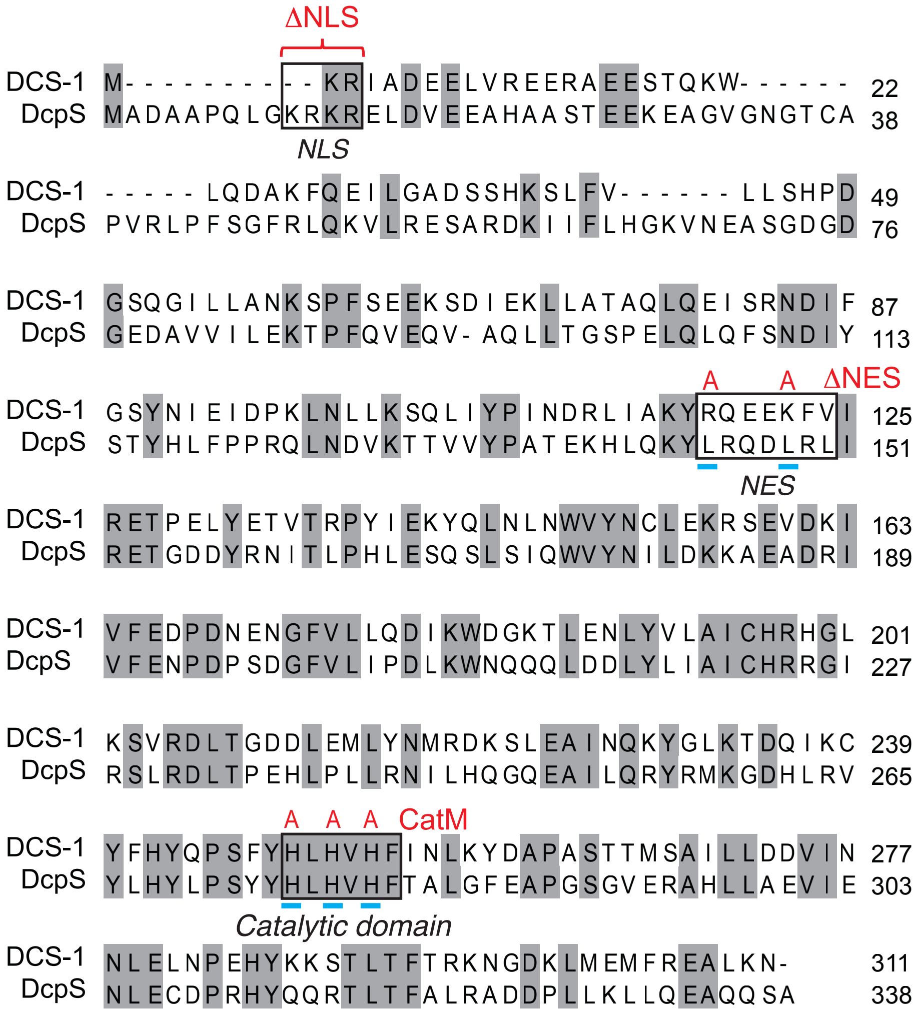

Figure S2. The amino acid sequence alignment between human DcpS and its *C. elegans* ortholog DCS-1. Mutations performed to produce DcpS-ΔNLS, DcpS-ΔNES and DcpS-CatM variants are shown.

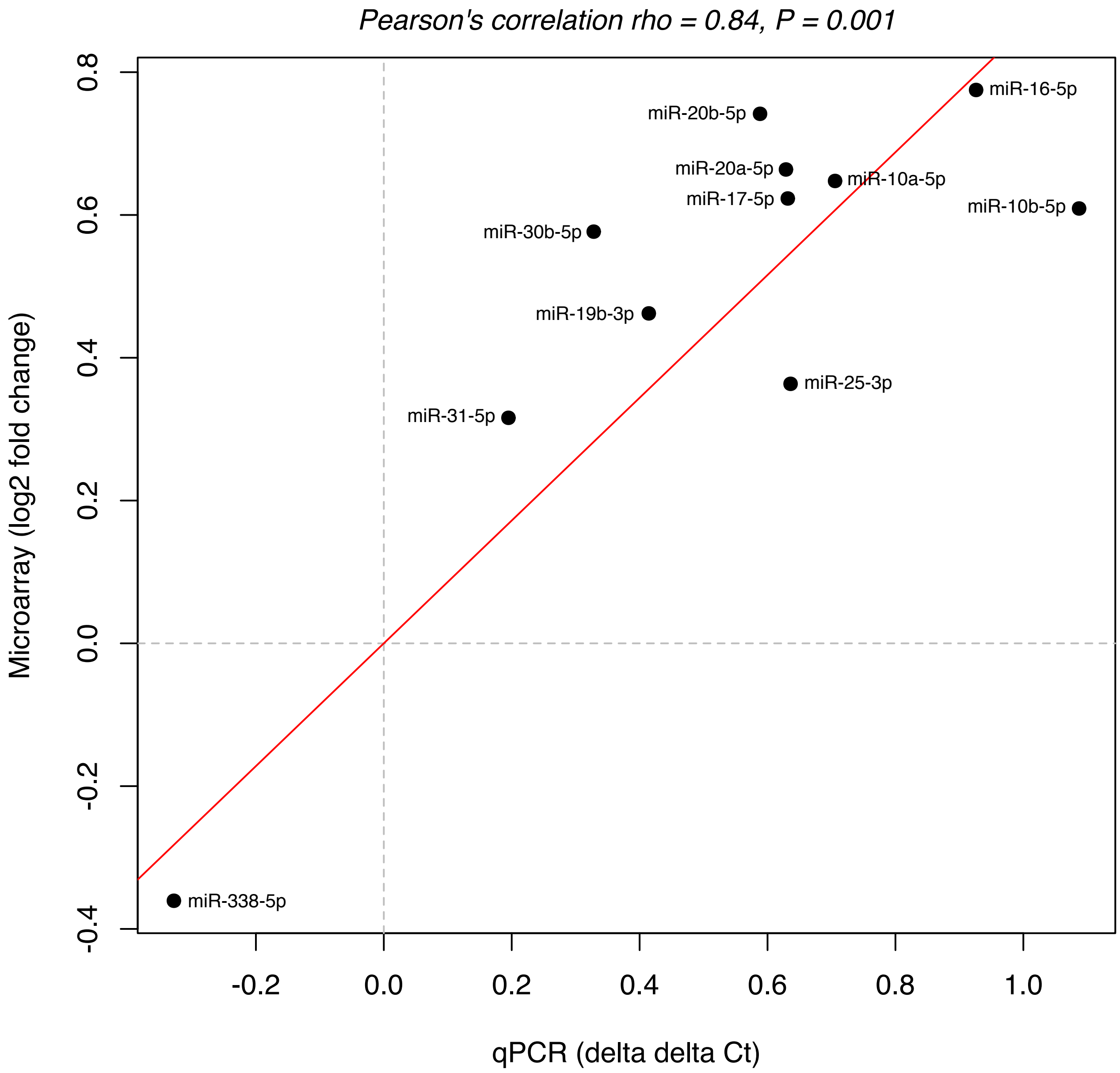

Figure S3: Validation of the microarray results using quantitative PCR.
The x-axis corresponds to delta delta Ct obtained using TaqMan assays for miRNAs indicated and normalized with the TaqMan assay of unaffected miRNA (miR-33b). The y-axis corresponds to the microarray results in log2 fold change. The red line represents a linear fit of the data points and the gray dashed lines show x = 0 and y = 0. We used a two-sided Pearson's correlation test to evaluate the agreement between qPCR and microarray results.

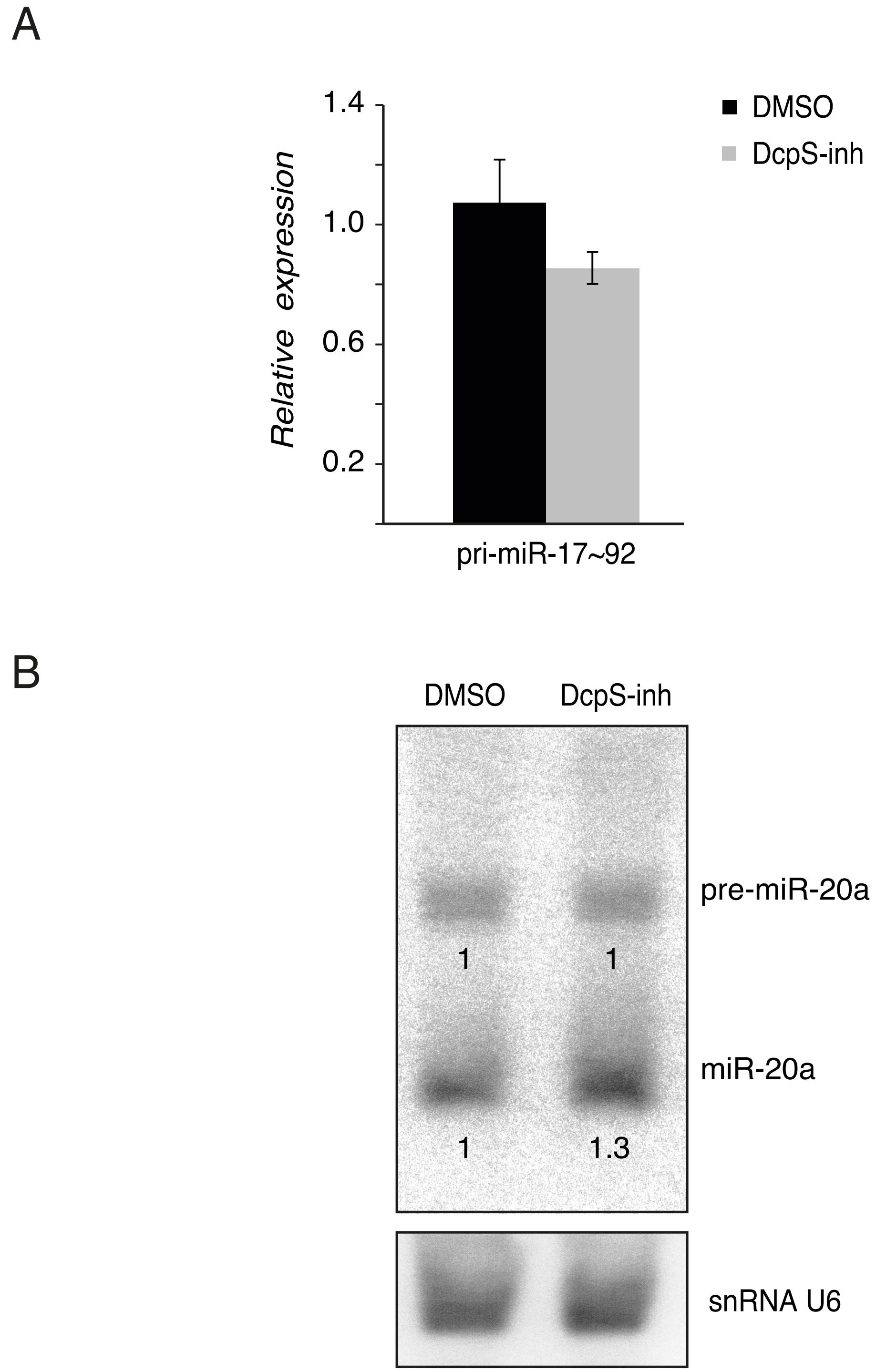

Figure S4: Inhibition of DcpS does not affect biogenesis and processing.
(**A**) Inhibition of DcpS does not affect pri-miRNA levels. Quantification of pri-microRNA cluster 17~92 coding for six miRNAs (miR-17, miR-18a, miR-19a, miR-20a, miR-19b and miR-92a) in cell treated with DcpS inhibitor compared to cell treated with DMSO. The relative expression of pri-miRNA levels was calculated using Delta delta Ct method, normalized to GAPDH mRNA. The graph represents the quantification of three independent experiments.The error bars represent standard errors and significance that were analyzed with a Student's t-test. (**B**) Detection by Northern blot hybridization of miR-20a and miR-20a precursor molecule from total RNA samples purified from cells treated with DMSO and DcpS inhibitor. The detection of snRNA U6 acted as a loading control. Relative quantification of precusor and mature miR-20a molecules are indicated under each band.
